# Real-time PCR detection of *Toxoplasma gondii* in tissue samples of wild boars (*Sus scrofa*) from southern Italy reveals high prevalence and parasite load

**DOI:** 10.1186/s13071-019-3586-5

**Published:** 2019-07-05

**Authors:** Mario Santoro, Maurizio Viscardi, Giovanni Sgroi, Nicola DʼAlessio, Vincenzo Veneziano, Roberta Pellicano, Roberta Brunetti, Giovanna Fusco

**Affiliations:** 1 Istituto Zooprofilattico Sperimentale del Mezzogiorno, 80055 Portici, Italy; 2 Department of Veterinary Medicine and Animal Productions, Università degli Studi di Napoli “Federico II”, 80137 Naples, Italy

**Keywords:** qPCR, Wildlife, *Toxoplasma gondii*, *Sus scrofa*, Food-borne disease, Zoonosis

## Abstract

**Background:**

Toxoplasmosis is a zoonotic parasitic disease caused by *Toxoplasma gondii*, a widespread protozoan in the phylum Apicomplexa. In Europe, several studies have demonstrated the presence of the parasite in tissues of wild boars (*Sus scrofa*), but no data exists on the *T. gondii* load in tissues which in turn may be an useful way to assess the infection risk for the consumer of wild boar meat.

**Methods:**

We sampled and tested a total of 472 tissue samples of brain, heart and masseter muscle from 177 wild boars from the Campania region of southern Italy by real-time PCR analyses for detection and quantification of *T. gondii*. The sensitivity and specificity of the method were calculated by ROC analysis curves.

**Results:**

PCR analysis revealed the presence of *T. gondii* in tissue samples of 78 out of 177 (44%) wild boars. In general, the brain presented the highest PCR prevalence (31%), followed by the heart (28.3%) and the masseter muscle (24.2%), with the highest estimated parasite numbers observed in the brain followed by the heart and masseter muscle. The PCR method showed an excellent discriminating ability for each of the examined tissues. According to the ROC analysis curves, the respective sensitivity and specificity were 99 and 100% for masseter muscle, 98 and 98% for brain and 96 and 98% for heart samples.

**Conclusions:**

The high prevalence of infection here detected suggests a widespread distribution of the parasite in the wildlife of the Campania region of southern Italy. The *T. gondii* burdens detected may potentially represent a source of infection for humans.

## Background

Toxoplasmosis is a zoonotic parasitic disease caused by *Toxoplasma gondii*, a widespread protozoan in the phylum Apicomplexa. It is considered one of the most successful parasitic pathogens considering the number of host species and percentage of animals infected globally, including approximately one-third of the human population infected. Toxoplasmosis is, overall, the third most common cause of human hospitalization due to food-borne infection [1, 2]. In Italy, it has been estimated that the overall average number of new infections per year among adults is 12,513, of which 92 are pregnant women [3].

The life-cycle of *T. gondii* includes three infectious stages: tachyzoites, which facilitate expansion during acute infection multiplying in several host cell types; bradyzoites, which maintain chronic infection and occur in tissue cysts; and sporozoites (contained within oocysts), which are shed in the environment within feces by felid definitive hosts [4]. Humans may become infected through the ingestion of food and water contaminated by sporulated oocysts or ingestion of cysts from infected tissues. Meat from domestic pigs has been shown to be an important source of infection for humans through the consumption of contaminated and infected fresh meat cuts and preparations [1–3, 5].

The increase in autochthonous wild boar (*Sus scrofa*) populations which have expanded their habitats throughout Europe, the recreational hunting of wild boars and, in turn, the increase in consumption of its meat, also increase the opportunities for the transmission of pathogens including toxoplasmosis from wild boars to humans [6]. According to Rostami et al. [7] the global seroprevalence of *T. gondii* among wild boars was 23% with a seropositivity rate of 26% in Europe. The wild boar is the main game food species consumed in Italy, reaching up to 4 kg/capita/year for hunter families [8]. The public perception that game meat is a sustainable, healthy and ecologically friendly product raises the potential for an increase in food-borne pathogen transmission associated with wildlife [8, 9]. A few studies have demonstrated the presence of *T. gondii* in different tissues of wild boars by PCR qualitative methods (positive/negative) [10–12], but no data exists on the quantification of parasite burden in its tissues which, in turn, may be a useful way to assess the infection risk for the consumer [13]. To our knowledge, this is the first time that the occurrence and parasite load of *T. gondii* in tissues of wild boars has been reported using a specific and sensitive real-time PCR assay.

## Methods

### Samples and DNA extraction

Between October and December 2018, adult wild boars (older than two years) were hunted under the framework of a control population program in the Campania region of southern Italy (D.G.R. no. 857/2015). During necropsies, brain, heart and masseter muscle samples of wild boars were collected and stored in individual vials at – 20 °C before genomic DNA extraction. The masseter muscle was chosen for sampling because this muscle in southern Italy is used to prepare a popular cured meat product named “guanciale” which is predominantly consumed uncooked.

For each sample, 1 g of tissue was individually homogenized by TissueLyser (Qiagen, Hilden, Germany), in sterile PBS buffer with two glass beads (5 mm). Using 200 µl of the homogenate, automated extraction of nucleic acid was performed by Qiasymphony SP/AS machinery using a commercial kit (QIAsymphony DSP Virus/Pathogen mini kit; Qiagen) according to the manufacturer’s protocol. As reference material, genomic DNA from *T. gondii* was obtained from the America type Culture Collection (ATCC 50174D LGC Standards Italy).

### Standard curve generation

A 193-bp fragment of *T. gondii* B1 gene was generated by PCR with the primers TOXO 1 and TOXO 2 according to Lin et al. [14], cloned into pGEM-T plasmid (pGEM-T Vector System I kit; Promega Corporation, Madison, WI, USA) and propagated in a JM109 *Escherichia coli* strain of high efficiency chemical competent cells, according to the manufacturer’s instructions. Plasmid DNA was purified using a Qiaprep Spin Miniprep Kit (Qiagen) and DNA integrity for the target was verified by capillary electrophoresis D5000 screen tape and reagent (Agilent Technologies, Santa Clara, California) on a 2200 Tapestation (Agilent Technologies) and confirmed by sequencing. The concentration of the extract was measured by Qubit Fluorometer (Thermo Fisher Scientific, Waltham, MA, USA) using Qubit dsDNA HS Assay kits. The plasmid copy number was calculated considering that the plasmid size (including the insert) was 3179 bp using the DNA/RNA Copy Number Calculator (http://www.endmemo.com/bio/dnacopynum.php). The appropriate dilutions were performed in order to produce aliquots of 10^10^ copies of DNA/10 µl of templates and frozen at – 80 °C for one use only. To generate the real-time PCR standard curves, 10-fold serial dilution of the *T. gondii* standard plasmid DNA, ranging from 10^1^ to 10^9^ copies of DNA/10 µl, were prepared for molecular quantifications. A standard curve was obtained by linear regression analysis of the threshold cycle (C_t_) value (y-axis) *versus* the log of the initial copy number present in each sample dilution (x-axis). PCR efficiency (E) was calculated as E = 10 (1/slope)^−1^ [14–16].

### Real-time PCR

Real-time PCR for detection and quantification of *T. gondii* B1 gene was performed following the methods by Lin et al. [14], with the exception that the final volume of reaction was 25 µl instead of 50 µl. In brief, template DNA was added to a reaction mixture containing 25 µl of 2× PCR universal master mix, 5 µl of the forward primer TOXO-F (5 µM, 5′-TCC CCT CTG CTG GCG AAA AGT-3′), 5 µl of the reverse primer TOXO-R (5 µM, 5′-AGC GTT CGT GGT CAA CTA TCG ATT G-3′) and 5 µl of TaqMan probe (2 µM, 6FAM-TCT GTG CAA CTT TGG TGT ATT CGC AG-TAMRA) in a final volume of 25 µl. The PCRs were performed with a GenAmp 5700 Sequence Detection System (Thermo Fisher Scientific). After initial activation of AmpliTaq Gold DNA polymerase at 95 °C for 10 min, 40 PCR cycles of 95 °C for 15 s and 60 °C for 1 min were performed. C_T_, indicative of the quantity of target gene at which the fluorescence exceeds a preset threshold, was determined. This threshold was defined as 20 times the standard deviation of the baseline fluorescent signal, i.e. the normalized fluorescent signal of the first few PCR cycles. After reaching the threshold, the sample was considered positive [14].

### Data analysis

The sensitivity and specificity of the real-time PCR (plus 95% confidence limits) were calculated by the receiver operator characteristic (ROC) analysis curve using IBM SPSS statistics v.25 software. An ANOVA test was used to look for the differences in mean parasite estimates (amount of copies/g) among the brain, heart and masseter samples. Significance was set at *P* < 0.05.

## Results

**Fig. 1:**
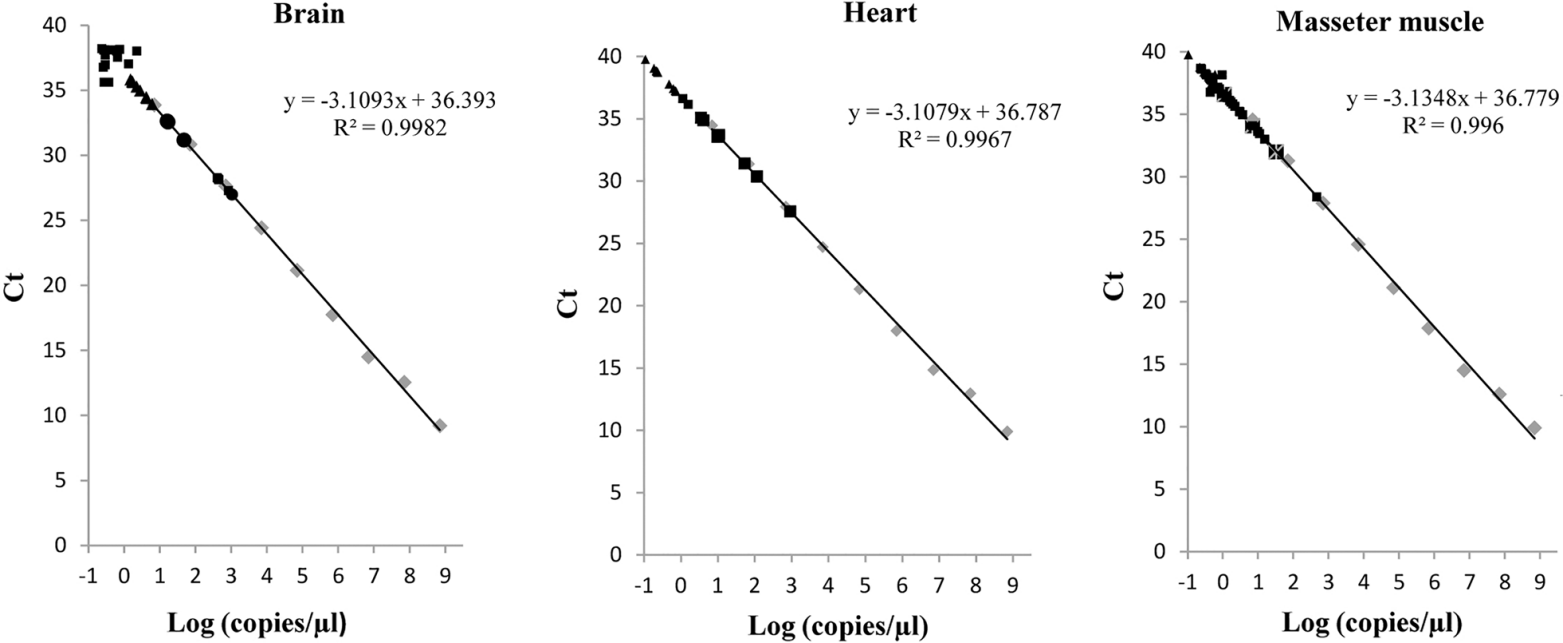
Standard curve for quantification of *Toxoplasma gondii* copies/g in brain, heart and masseter muscle samples of wild boars from the Campania region of southern Italy

We obtained samples from 177 wild boars and performed real-time PCR analyses on a total of 472 tissue samples including 141 from the brain, 166 from the heart and 165 from the masseter muscle. In general, PCR analysis revealed the presence of *T. gondii* in the tissues of 78 out of 177 (44%) wild boars; 36 wild boars tested positive in a single tissue, 31 tested positive in two tissues and 11 tested positive in three tissues. Table 1 shows the prevalence of infection and number of *T. gondi* copies/g for each of the examined tissues. Figure 1 shows the standard curve for the quantification of *T. gondii* copies in the brain, heart and masseter muscle samples, respectively. In general, the brain samples presented the highest PCR prevalence (31%), followed by heart samples (28.3%) and masseter muscle samples (24.2%). C_t_ values ranged from 26.99 to 39.77 with the lowest C_t_ values corresponding to the highest parasite estimates observed in the brain followed by the heart. The amount of copies/g among the positive samples ranged from 84 to 837,600. Most of *T. gondii* positive samples (48%) showed a number of copies ranging from > 0.1 to < 1; 15.8% showed a number of copies ranging from > 1 to < 10; 5.6% showed a number of copies ranging from > 10 to < 100; 3.9% showed a number of copies ranging from > 100 to < 1000; and only one brain sample (0.5%) showed a number of copies > 1000. Higher prevalence of *T. gondii* detection for masseter samples was observed when the number of parasite copies ranged from > 1 to < 10.

**Table 1:** Results of real-time PCR listing the minimum, maximum and mean values of numbers of *Toxoplasma gondii* copies per gram tissue of the brain, heart and masseter muscle of wild boars from southern Italy

Tissues	*N*	RT-PCR positive *n* (%)	Minimum value (copies/g)	Maximum value (copies/g)	Mean value (copies/g)
Brain	141	44 (31.2)	0.0019 × 10^5^	8.38 × 10^5^	4.19 × 10^5^
Heart	166	47 (28.3)	0.00088 × 10^5^	7.37 × 10^5^	3.68 × 10^5^
Masseter muscle	165	40 (24.2)	0.00084 × 10^5^	3.68 × 10^5^	1.8 × 10^5^

**Fig. 2:**
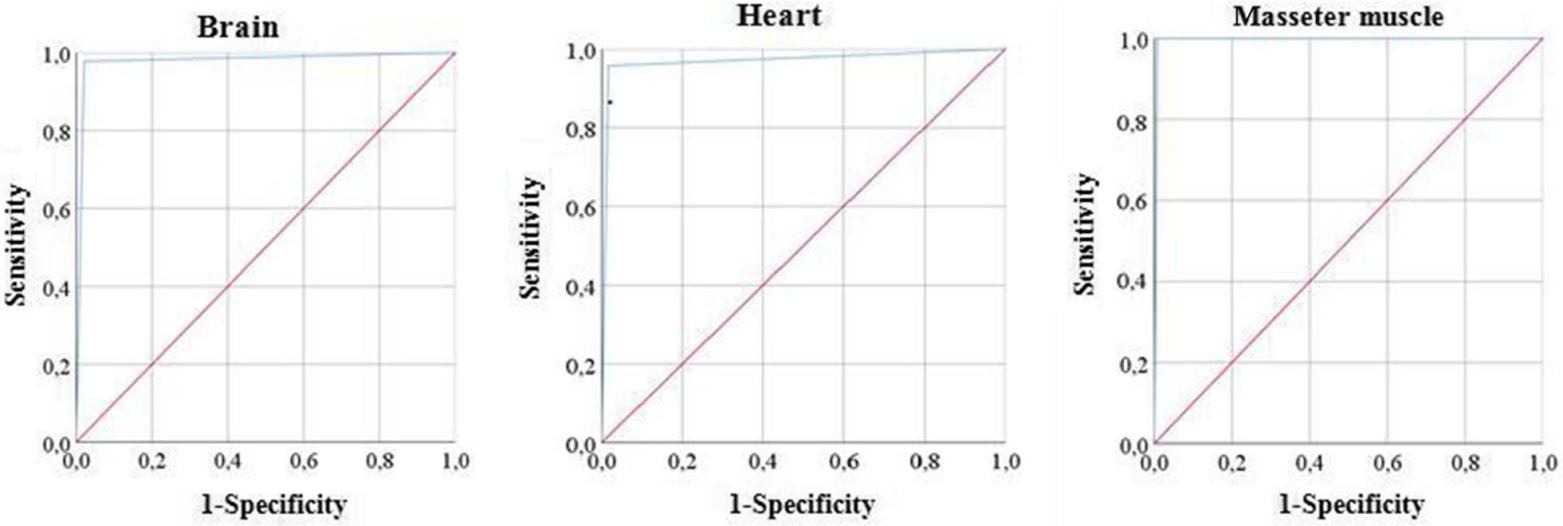
ROC curve analysis sensitivity and specificity for real-time PCR performed for brain, heart and masseter muscle samples of wild boars from the Campania region of southern Italy

According to the ROC analysis curve, the respective sensitivity and specificity were 99% (95% CI: 96–99%) and 100% (95% CI: 90–100%) for the masseter muscle, 98% (95% CI: 88–99%) and 98% (95% CI: 93–99%) for the brain, and 96% (95% CI: 85–99%) and 98% (95% CI: 94–99%) for the heart samples. The estimated area under the curve was 0.97 for the brain and heart and 0.99 for the masseter muscle (Fig. 2). Variance analysis tests showed no significant statistical differences among the tissues studied (*F*_(2,468)_ = 2.065; *P* = 0.128).

## Discussion

To our knowledge, this study reports the first quantitative molecular data on the presence of *T. gondii* in tissue samples of wild boars. ROC curve analysis was used to test the sensitivity and specificity of the real-time PCR test. ROC curve analysis for each of the examined tissues, corresponding to a progressively greater discriminant capacity of diagnostic tests, was located progressively closer to the upper left-hand corner in the ROC space (Fig. 2). The estimated area under the ROC curves that summarizes the entire location of the ROC curve showed an excellent discriminating ability since the values of the area for the *Toxoplasma* real-time PCR were close to 1.0 ranging from 0.99 in the masseter muscle to 0.97 in both the brain and heart samples [17].

The DNA of *T. gondii* was found from tissues of 44% of the wild boars showing both high prevalence and parasite load. Concerning the prevalence, due to the methodology used, we have only been able to compare our results with those of Ferroglio et al. [10]; these authors found a prevalence of 16.19% in wild boars from the western Italian Alps [10]. Prevalence here recorded was almost three times higher than that previously recorded in northern Italy [10]. Differences in prevalence of infection by *T. gondii* have been related to several factors including climate characteristics of the host habitat, and the size and weight of the host species, which are usually correlated with the duration of its life, and the diet and feeding behavior of the host species. The prevalence of infection is often lower in herbivores than in omnivores and carnivores due to the cumulative efficacy of the predator-prey cycle of the parasite [10]. The overall prevalence of infection detected here suggests a widespread distribution of the parasite in the wildlife of the Campania region of southern Italy.

Concerning the distribution of *T. gondii* according to the tissues studied, the highest prevalence was found in the brain (31.2%), followed by the heart (28.3%) and masseter muscle (24.2%). These results are in agreement with previous studies identifying the brain followed by the heart as the most important tissue targets for *T. gondii* infection when pigs are infected experimentally with oocysts [5, 13, 18, 19]. The present study showed no significant differences among the three tissues examined when parasite load was considered. Our results contrast with those of previous studies on experimentally infected pigs [13, 19]. Juránková et al. [19] found that the parasite load in the brain was significantly higher than in heart samples. In contrast, Gisbert-Algaba et al. [13] found that the parasite load in the heart was significantly higher than in brain samples. The usage of different oocyst doses in those experimental infections was considered as a plausible explication for such differences [13]. Moreover, in pig tissues, *T. gondii* load has been shown to be strain dependent showing a pronounced clearance when a hybrid type I/II strain is used rather than a classical type II strain for experimental infection [20, 21]. In addition, it has been recently observed that pigs experimentally infected with oocysts had a significantly higher parasite load than pigs infected with tissue cysts [13]. Because we studied wild boars collected in the wild, the source of the host infection (oocysts or tissue cysts) remains unknown and may be only speculated. Wild boars may come into contact with a wide range of prey due to their scavenging habits (including birds, rodents and other mammals), perhaps more likely ingesting tissue cysts and acquiring several *T. gondii* genotypes, including atypical ones [10, 11]. In contrast, infection with sporulated oocysts defecated by cats is unlikely to occur in the remote mountainous areas of southern Italy where the wild boars were hunted.

Regarding the parasite load among the different tissues, it has been observed in experimental studies using rodents and livestock animals that the parasite load may vary depending on time since infection, with a significantly higher parasite load occurring in the brain, liver and blood only at the onset, and with the parasite load in the heart and skeletal muscles increasing over time [19, 22]. On the basis of the results of such studies, we may hypothesize that in wild boar muscles, the *T. gondii* load may increase over time. Moreover, since *T. gondii* infection does not cause a fatal infection in suids, it is plausible to think that the tissue cysts may remain viable in wild boar muscles for several months, increasing in number over time and continuing the source of infection in the environment [5, 23].

## Conclusions

Our results suggest that the real-time PCR is a specific, sensitive and easy to perform method for *T. gondii* detection in different tissue samples of wild boars. The preferred site for *T. gondii* in wild boars was the brain, which was the tissue with the highest parasite load; therefore, it appears to be the most appropriate organ for parasite detection and isolation of *T. gondii* in this host. The heart also showed a high parasite load, and may represent a potential source of infection for humans as it is frequently consumed in southern Italy. The lowest parasite load was detected in masseter muscles; however, since this muscular tissue is used to prepare a popular cured meat product (guanciale) which is predominantly consumed uncooked, it may potentially represent an important source of *T. gondii* infection for humans.

## Data Availability

The data supporting the conclusions of this article are included within the article.

## Abbreviation

PCR: polymerase chain reaction
