# Correction to: Real-time PCR detection of *Toxoplasma gondii* in tissue samples of wild boars (*Sus scrofa*) from southern Italy reveals high prevalence and parasite load

**DOI:** 10.1186/s13071-021-05004-w

**Published:** 2021-10-11

**Authors:** Mario Santoro, Maurizio Viscardi, Giovanni Sgroi, Nicola DʼAlessio, Vincenzo Veneziano, Roberta Pellicano, Roberta Brunetti, Giovanna Fusco

**Affiliations:** 1 Istituto Zooprofilattico Sperimentale del Mezzogiorno, 80055 Portici, Italy; 2 Department of Veterinary Medicine and Animal Productions, Università degli Studi di Napoli “Federico II”, 80137 Naples, Italy

## Correction to: Parasites Vectors 12:335 (2019) https://doi.org/10.1186/s13071-019-3586-5

Following publication of the original article [1], an error was identified in the paragraph Real-time PCR (from line 4 to line 12) of the Methods. The correct information is given below:

”In brief, 5 µl of template DNA was added to a reaction mixture containing 12.5 µl of 2× PCR universal master mix (Thermo Fischer Scientific), 2.5 µl of the forward primer TOXO-F (5 µM, 5ʹ-TCCCCTCTGCTGGCGAAAAGT-3ʹ) (IDT), 2.5 µl of the reverse primer TOXO-R (5 µM, 5ʹ-AGCGTTCGTGGTCAACTATCGATTG-3ʹ) (IDT), and 2.5 µl of TaqMan probe (2 µM, 6FAM TCTGTGCAACTTTGGTGTATTCGCAG-TAMRA) (Thermo Fischer Scientific) in a final volume of 25 µl.”

The original article has since been updated.

The authors apologize for any inconvenience caused.
